# C-peptide is a predictor of telomere shortening: A five-year longitudinal study

**DOI:** 10.3389/fendo.2022.978747

**Published:** 2022-08-18

**Authors:** Racha Ghoussaini, Hani Tamim, Martine Elbejjani, Maha Makki, Lara Nasreddine, Hussain Ismaeel, Mona P. Nasrallah, Nathalie K. Zgheib

**Affiliations:** ^1^ Faculty of Medicine, School of Medicine, American University of Beirut Medical Center, Beirut, Lebanon; ^2^ Faculty of Medicine, Clinical Research Institute, American University of Beirut Medical Center, Beirut, Lebanon; ^3^ Faculty of Medicine, Department of Internal Medicine, American University of Beirut Medical Center, Beirut, Lebanon; ^4^ College of Medicine, Alfaisal University, Riyadh, Saudi Arabia; ^5^ Faculty of Agricultural and Food Sciences, Department of Nutrition and Food Sciences, American University of Beirut, Beirut, Lebanon; ^6^ Vascular Medicine Program, American University of Beirut, Beirut, Lebanon; ^7^ Faculty of Medicine, Department of Internal Medicine, Division of Cardiology, American University of Beirut, Beirut, Lebanon; ^8^ Faculty of Medicine, Department of Internal Medicine, Division of Endocrinology, American University of Beirut Medical Center, Beirut, Lebanon; ^9^ Faculty of Medicine, Department of Pharmacology and Toxicology, American University of Beirut, Beirut, Lebanon

**Keywords:** telomere length, C-peptide, aging, metabolic, insulin resistance, predictor, telomere shortening

## Abstract

**Aim:**

Relative telomere length (RTL) predicts the development of many age-related diseases. Yet, few studies have evaluated their longitudinal effect on RTL. We investigated longitudinally the association between cardiometabolic risk factors and RTL.

**Methods:**

This was a longitudinal study with a 5-year follow-up period, based on data collected in 2014 and 2019. Of 478 participants in 2014, 198 consented to be followed-up in 2019. The associations between RTL and risk factors were analyzed using t-test, ANOVA or simple linear regression as applicable.

**Results:**

RTL was significantly shortened after 5 years (P<0.001). Older age (P=0.018) and gender (P=0.05) were significantly associated with shorter RTL at follow-up. Higher baseline C-peptide correlated with shorter RTL (P=0.04) and shortening of RTL (P=0.03) after 5 years. Multivariate linear regression including both age and gender revealed a significant trend for C-peptide and change in RTL after 5 years (P=0.04). Interestingly, there was a trend of shorter RTL at follow-up with diabetes, though the findings were not statistically significant.

**Conclusions:**

Higher C-peptide level contributes to telomere shortening over time, suggesting that metabolic dysregulation may play a role in early aging. Further understanding of this relationship and addressing high C-peptide levels can be important to prevent premature aging.

## Introduction

Telomeres are repetitive DNA sequences that shelter the linear ends of chromosomes in order to prevent DNA damage (1). They are added by the telomerase enzyme to prevent the loss of coding nucleotides, a process that normally occurs with each cell division due to incomplete replication of the 5’end of the DNA lagging strand (2). As a result, with each replicative cell division, telomeres will shorten, and this process has been correlated with cellular senescence (3). In addition to that, telomere shortening can be accelerated in the setting of disease conditions characterized by increased inflammation, which makes it a measurable marker of inflammation and oxidative stress (4).

Many studies have shown significant correlations between certain metabolic conditions associated with aging and relative telomere length (RTL) over time. As such, telomere length, as measured in leukocytes, is now widely recognized as a marker for cellular aging and as a clinical indicator for comorbidity and disease (2). For instance, a longitudinal cohort study that included 2848 adult men and women from the Netherlands recruited between 2004 and 2007, showed that a shorter baseline RTL was associated with worsening of all components of the metabolic syndrome at 2- and 6-years follow-up (5). Similarly, a study conducted on a cohort of 12792 Mexican American participants aged 20 to 85 years and followed annually, showed a doubling in the incidence of diabetes over time, between the lowest and highest quartiles of RTL at baseline (6). These findings support the role of RTL as a predictor of disease and morbidity, yet they only highlight a unidirectional relationship between shortening of RTL and the development of comorbidities years later. However, the relationship between RTL and comorbidities is complex and bidirectional. Baseline comorbidities such as obesity, hypertension, diabetes, and dyslipidemia could influence the change in RTL over time. Such a relationship would constitute a potential driver of a vicious metabolic cycle whereby a short RTL will lead to worsening metabolism, that in turn will accelerate RTL shortening even further (7).

Thus, it becomes important to better characterize the relationship between these cardiometabolic risk factors and their effect on RTL. There are few longitudinal studies that demonstrated the impact of such risk factors on RTL at follow-up, and on the rate of telomere shortening. For instance, a study on the same cohort from the Netherlands assessed baseline cardiometabolic risk factors and rate of RTL shortening over time in 1808 participants; it showed that a higher baseline waist circumference, lower HDL-cholesterol, and higher glucose levels all correlated with shorter telomere length at 6-year follow-up. This study also revealed that a larger increase in waist circumference over time was associated with significantly shorter RTL at follow-up (8). A separate longitudinal cohort study conducted a decade earlier in 1998 on 8074 participants ages 28-75 years from the Netherlands, and followed over 6.5 years, estimated the average loss in RTL to be 0.47 ± 0.16 annually. However, this rate was 3 times higher among active smokers, and was also accelerated in the presence of higher baseline waist, higher glucose levels and lower HDL-cholesterol (9).

Even though the above metabolic correlations allude to it, there are no studies reporting longitudinally on the presence of diabetes, a major cardiometabolic risk factor, and its effect on RTL. Moreover, there are few longitudinal reports on markers of dysglycemia, such as insulin and C-peptide levels. A longitudinal measure of both RTL and insulin resistance conducted over 12 years on 338 adult twin pairs found RTL to predict the development of insulin resistance, but not vice versa (10). The main driver of this relationship was insulin level and not glucose. Being strongly associated with aging, we were interested in determining longitudinally the effect of diabetes and other markers of dysglycemia on RTL over time (11). We have previously reported in a cross-sectional study in the Lebanese population an association between telomere length and risk factors such as a wider waist circumference and hypertension (12).

The purpose of the current study is to determine longitudinally whether the health profile or the presence of cardiometabolic risk factors influence the change in RTL over time in a sample of Lebanese individuals. This is with the aim of better characterizing the bidirectional relationship between RTL and these chronic conditions.

## Materials and methods

The current study builds on subjects, data and blood samples collected in 2014 with a follow up recruitment, data and blood samples collection 5 years later in 2019. The study was approved by the Institutional Review Board at the American University of Beirut with participants signing an informed consent form on both recruitment stages.

### 2014 baseline study

A detailed description of the study design has been reported elsewhere (12). Briefly, 501 adult Lebanese men and women, aged 18 years old and above, were recruited between February and June 2014. They were all residing in Greater Beirut. The objective was to determine Bisphenol A levels among residents of Greater Beirut (13).

Baseline data collection consisted of face-to-face interviews, anthropometric measurements, and blood withdrawal for laboratory tests. Data on demographic and socioeconomic characteristics namely age, gender, marital status, education, occupation, crowding index, and monthly income per family were collected. Lifestyle factors namely current smoking status including regular cigarettes and narguileh (waterpipe), alcohol intake, caffeine intake and physical activity, based on the short version of the International Physical Activity Questionnaire (IPAQ 2004), as well as sleep patterns were also recorded. Sleep apnea score was calculated based on the Berlin index (14). Anthropometric measurements included weight and height using calibrated scales, and waist circumference based on standardized protocols. A calibrated sphygmomanometer was used to determine both systolic and diastolic blood pressure. Medical history for chronic diseases that is type 2 diabetes, hypertension, and dyslipidemia as well as the drug intake for these conditions were collected as well.

Whole blood drawn into an EDTA tube was stored at -80 °C for DNA isolation and RTL measurement. The rest was kept at -20 °C for laboratory measurements which included fasting glucose, hemoglobin A1c (HbA1c), insulin, C-peptide, lipid profile, cortisol, and C-reactive protein (CRP). ‘Definite diabetes’ variable was computed for participants who either had a known history of diabetes or had both a fasting blood glucose (FBG) ≥126 mg/dL and HbA1c ≥6.5% (48 mmol/mol) (15). ‘Definite hypertension’ was computed for participants who had a known history of hypertension or had an abnormal blood pressure reading upon recruitment (systolic blood pressure > 140 mmHg or diastolic blood pressure > 90 mmHg) (16), while ‘Central obesity’ was defined as waist circumference above the cutoffs of 80 cm for women and 94 cm for men, as per the recommendation to refer to European data for Arab populations by the International Diabetes federation (17).

### Measurements

Relative telomere length and laboratory values were measured as previously described with brief details shown below (12).

#### Relative telomere length

Total DNA was isolated from circulating white blood cells from peripheral venous blood using Qiagen kit (Qiagen, USA), at a concentration of 10 ng/ul, and stored at -20 °C. Telomere length was then measured by quantitative real-time polymerase chain reaction (RT-qPCR), through telomere (tel) and single copy gene (human beta-globin-hbg) amplification separately, on CFX384 Touch Real-Time PCR Detection System from BIO-RAD. This method was detailed by Cawthon in 2002 (18), with few adjustments made in 2009 (19).

RTL was calculated as per the formula described by Pfaffl 2001 (20), which accounts for different plate efficiencies.

#### Laboratory tests

Laboratory tests were performed with the following methodologies: fasting glucose by the enzymatic method (Cobas 6000, Roche), HbA1C by HPLC (Bio-Rad), insulin by radioimmunoassay (Cisbio), C-peptide by radioimmunoassay (Cisbio), and cortisol by radioimmunoassay (Cisbio). Levels of triglycerides, HDL, LDL, total cholesterol, and CRP were measured using Vitros 350 analyzer (Ortho Clinical Diagnostics, Johnson and Johnson).

### 2019 follow up study

Five years after the baseline study, and between February and May 2019, participants who consented to be contacted again were called by phone for the purpose of scheduling a follow-up visit for similar procedures performed in 2014. The sample that was available for follow-up consisted of 478 participants, out of which 198 agreed to be part of the study.

A detailed description of the follow up design has been recently reported elsewhere (21). Briefly, participants underwent face-to-face interview, anthropometric measurements, and laboratory studies. All previously (2014) collected demographics, socioeconomic information lifestyle factors, and concomitant diseases were similarly collected in the follow up study. The same applies for the anthropometric and blood pressure measurements and blood collection.

Total DNA was extracted from leucocytes of peripheral venous blood, and RTL was measured by amplifying telomere and single copy gene separately, using quantitative real-time PCR with the same analyses and quality control parameters as described for the 2014 baseline study in the same laboratory (12). The follow-up results were adjusted for systematic differences caused by the use of different reference samples.

### Statistical analysis

Data were entered into SPSS and a *P* value <0.05 was used to indicate statistical significance. Variables on the presence of metabolic syndrome and the atherosclerotic vascular disease risk were computed as previously described (12). Results are presented as means ± standard deviation (SD) for continuous variables and as percentages for categorical variables.

The difference between mean RTL at baseline versus at follow up was compared using paired t-test. The associations of baseline demographics as well as lifestyle factors at initial recruitment (2014) with RTL at follow-up (2019) and change in RTL (defined as the difference between RTL at follow-up and RTL at recruitment) were first evaluated using Student t-test or One way ANOVA and simple linear regression using Pearson coefficient (r) as applicable. The same tests were conducted for the associations between baseline cardiometabolic factors factor, RTL and change in RTL at follow-up. Afterwards, morbidities that were found to be significantly associated with RTL or change in RTL at follow-up were assessed using a multivariate linear regression model including both age and gender. Results of the multivariate analyses are reported as beta coefficient (β) with 95% confidence intervals (CI).

## Results

DNA for RTL measurement was not available for two of the participants, hence the final sample size was 196. RTL was significantly shortened after 5 years follow up with a mean ± SD of 1.48± 0.85 in 2014 compared to 1.16 ± 1.00 in 2019 (*P*<0.001 with paired t-test). Furthermore, baseline creatinine (from the 2014 visit) of the 196 participants that were available for the 5-year follow-up was calculated as 0.75 ± 0.21, with only 5 participants with a GFR below 60 mL/min/1.73 m^2^ (6.3%), none of whom had a GFR of below 30 mL/min/1.73 m^2^.

### Baseline characteristics and RTL

**Table 1** depicts the association of baseline characteristics and lifestyle factors at initial recruitment (2014) with RTL at follow-up 5 years later, as well as the change in RTL in 2019 compared to 2014.

**Table 1:** Description and association of baseline characteristics and lifestyle at initial recruitment in 2014 with RTL at follow up in 2019 and ΔRTL.

			RTL at follow-up		ΔRTL	
Variables in 2014		Total N=196	Correlation (r) Mean ±SD	p-value	Correlation (r) Mean ±SD	p-value
**Age (years)**	**Mean±SD**	46.88±13.34	-0.168	**0.018**	-0.060	0.407
	**<40**	58 (29.6)	1.42±1.56	0.07	-0.31±1.56	0.559
	**40-60**	113 (57.9)	1.07±0.61		-0.26±0.80	
	**>60**	25 (12.8)	1.02±0.61		-0.53±1.14	
**Gender**	**Female**	125(63.8)	1.27±1.17	**0.05**	-0.27±1.23	0.487
	**Male**	71 (36.2)	0.98±0.55		-0.38±0.87	
**Marital Status**	**Married**	146 (74.5)	1.15±1.08	0.54	-0.34±1.13 -0.24±1.51 -0.17±0.65	0.724
	**Single**	21 (10.7)	1.38±0.73			
	**Other**	29 (14.8)	1.07±0.70			
**Income**	**<600$**	67 (34.5)	1.04±0.63	0.57	-0.35±0.96	0.377
	**600-999.9$**	79 (40.7)	1.21±1.34		-0.31±1.39	
	**≥1000$**	37 (19.1)	1.21±0.76		-0.40±0.81	
	**I don’t know/no answer**	11 (5.7)	1.43±0.74		0.24±0.61	
**Education**	**No schooling or primary school**	76 (39.0)	1.00±0.62	0.19	-0.46±0.94	0.303
	**Intermediate school**	60 (30.8)	1.37±1.52		-0.26±1.38	
	**Secondary school or technical diploma**	44 (22.6)	1.14±0.67		-0.08±0.63	
	**University degree**	15 (7.7)	1.24±0.57		-0.47±1.74	
**Crowding Index**	**Mean±SD**	1.56±0.80	-0.067	0.353	0.024	0.741
**Smoker**	**Never**	44 (22.4)	1.11±0.66	0.87	-0.42±0.90	0.75
	**Former**	27 (13.8)	1.23±0.69		-0.22±0.91	
	**Current**	125 (63.8)	1.18±1.15		-0.30±1.23	
**Cigarette smoker**	**Never**	97 (49.5)	1.23±1.28	0.68	-0.32±1.29	0.66
	**Former**	22 (11.2)	1.12±0.48		-0.11±0.50	
	**Current**	77 (39.3)	1.10±0.65		-0.36±1.01	
**Narguileh (waterpipe) smoker**	**Never**	111 (56.6)	1.14±0.67	0.88	-0.28±0.81	0.80
	**Former**	29 (14.8)	1.19±0.68		-0.28±0.92	
	**Current**	56 (28.6)	1.22±1.56		-0.40±1.63	
**Current Alcohol Drinker**	**No**	168 (85.7)	1.69±0.94	0.502	-0.28±1.07	0.430
	**Yes**	28 (14.3)	1.60±1.04		-0.46±1.35	
**Coffee Drinker**	**No**	39 (19.9)	1.19±0.62	0.858	-0.41±1.33	0.544
	**Yes**	157 (80.1)	1.16±1.07		-0.28±1.06	
**BMI (kg/m^2^)**	**Mean±SD**	29.99±5.87	-0.048	0.508	-0.059	0.412
**BMI (kg/m^2^)- categorical**	**<30**	100 (51.0)	1.16±1.22	0.987	-0.32±1.22	0.904
	**≥30**	96 (49.0)	1.17±0.70		-0.30±1.01	
**Waist Circumference (cm)**	**Mean±SD**	97.85±17.23	-0.026	0.714	0.009	0.901
	Within normal per gender*	38 (19.4)	1.23±0.61	0.69	-0.15±0.68	0.31
	**Above normal per gender**	158 (80.6)	1.16±1.09		-0.35±1.20	
**Body Fat (Kg)**	**Mean±SD**	30.26±11.69	0.075	0.296	0.026	0.722
**Muscle Mass (Kg)**	**Mean±SD**	26.82±6.67	-0.091	0.207	-0.048	0.505
**Levels of Physical Activity**	**Low-intensity activity**	91 (46.4)	1.23±1.28	0.271	-0.39±1.40	0.355
	**Moderate-intensity activity**	72 (36.7)	1.02±0.50		-0.31±0.73	
	**High-intensity activity**	33 (16.8)	1.31±0.89		-0.06±0.87	
**Physical Activity**	**None**	28 (14.3)	0.98±0.52	0.293	-0.40±0.76	0.647
	**Any**	168 (85.7)	1.20±1.05		-0.29±1.16	
**Number of hours sleep per night on weekdays**	**<6 hours**	76 (38.8)	1.14±0.69	0.10	-0.23±0.80	0.37
	**6-8 hours**	92 (46.9)	1.09±0.59		-0.43±1.14	
	**>8 hours**	28 (14.3)	1.54±2.14		-0.15±1.68	
**Number of hours sleep per night on weekend**	**<6 hours**	59 (30.1)	1.18±0.76	0.51	-0.20±0.85	0.46
	**6-8 hours**	80 (40.8)	1.08±0.56		-0.43±1.14	
	**>8 hours**	57 (29.1)	1.28±1.56		-0.27±1.33	
**OSA score (berlin score)**	**Low risk**	109 (65.3)	1.24±1.22	0.58	-0.29±1.23	0.97
	**High risk**	58 (34.7)	1.15±0.74		-0.28±1.03	

*Gender normal cutoffs for waist circumference were cutoffs of 80 cm for women and 94 cm for men. Bolded P-values are statistically significant as they are less or equal to 0.05.

There was a significant association between gender and RTL at follow-up whereby males had a shorter RTL as compared to females (0.98±0.55 versus 1.27±1.17, *P =* 0.05). Similarly, older age was associated with shorter RTL at follow-up (r= -0.168, *P* = 0.018). RTL was otherwise not associated with socioeconomic indicators such as income, education or crowding index; nor with lifestyle factors such as coffee drinking, alcohol consumption, smoking, or physical activity. Finally, RTL did not correlate with any measures of obesity as reflected by BMI, waist circumference, or body fat mass.

### Baseline cardiometabolic risk factors and RTL

**Table 2** describes the association between baseline cardiometabolic risk factors (and related laboratory parameters) and RTL at follow-up 5 years later, as well as the change in RTL in 2019 as compared to 2014. A higher C-peptide level was significantly correlated with shorter RTL at follow-up (r= -0.14, *P* = 0.04). As for the change in RTL (ΔRTL), the only significant result was observed with C-peptide. For instance, there was again a negative correlation whereby a higher C-peptide level was significantly associated with a negative change in RTL, meaning shortening of RTL at follow up (r = -0.15, *P* = 0.03). There was an interesting trend between diabetes and RTL at follow-up, though it did not reach significance. For instance, participants with definite diabetes in 2014 had shorter RTL at follow up when compared to those who did not have diabetes (0.89±0.53 as compared to 1.22±1.06 respectively, with a *P* = 0.09).

**Table 2:** Association of cardiometabolic risk factors and laboratory values in 2014 with RTL at follow up in 2019 and ΔRTL.

Cardiometabolic risk factors and laboratory values in 2014	All sample N=196	RTL at follow-up		ΔRTL	
		Correlation (r) mean±SD	p-value	Correlation (r) mean±SD	p-value
**Diabetes**					
**Definite Diabetes**			0.09		0.30
No	163 (83.2)	1.22±1.06		-0.27±1.12	
Yes	33 (16.8)	0.89±0.53		-0.49±1.08	
**Self-reported diabetes or hyperglycemia diagnosis**			0.16		0.88
No	169 (86.2)	1.21±1.05		-0.30±1.15	
Yes	27 (13.8)	0.93±0.54		-0.33±0.94	
**Diabetes treatment**			0.24		0.84
No	169 (86.2)	1.20±1.05		-0.31±1.14	
Yes	27 (13.8)	0.95±0.53		-0.27±0.95	
**Fasting serum glucose (mg/dL)**			0.37		0.138
<100 – normal	90 (45.9)	1.10±0.55		-0.44±0.96	
≥100 – abnormal	106 (54.1)	1.22±1.26		-0.20±1.23	
**Insulin (μIU/mL), Mean (±SD)**	28.95±11.62	-0.063	0.415	-0.046	0.558
**HbA1c (%), Mean (±SD)**	5.9±1.2	-0.098	0.170	-0.021	0.768
**C peptide (ng/dL), Mean (±SD)**	3.18±1.37	-0.146	**0.044**	-0.154	**0.033**
**Hypertension (HTN)**					
**Definite HTN**					
No	127 (64.8)	1.20±1.12	0.51	-0.36±1.20	0.40
Yes	69 (35.2)	1.10±0.71		-0.22±0.95	
**Self-reported HTN diagnosis**			0.26		0.94
No	159 (81.1)	1.20±1.07		-0.31±1.20	
Yes	37 (18.9)	1.00±0.57		-0.30±1.26	
**HTN treatment**			0.17		0.79
No	161 (82.1)	1.21±1.07		-0.30±1.14	
Yes	35 (17.9)	0.96±0.53		-0.35±1.02	
**Systolic Blood Pressure (mm/Hg), Mean (±SD)**	121.97±19.05	-0.120	0.093	0.007	0.921
**Diastolic Blood Pressure (mm/Hg), Mean (±SD)**	75.73±10.28	0.008	0.908	0.062	0.388
**Dyslipidemia**					
**Self-reported dyslipidemia diagnosis**			0.83		
No	146 (74.5)	1.17±1.09		-0.36±1.20	0.27
Yes	50 (25.5)	1.14±0.65		-0.16±0.80	
**Dyslipidemia treatment**			0.11		0.90
No	156 (79.6)	1.22±1.09		-0.30±1.18	
Yes	40 (20.4)	0.94±0.08		-0.33±0.85	
**HDL (mg/dL), Mean (±SD)**	49.38±13.80	-0.052	0.465	-0.058	0.420
**LDL (mg/dL), Mean (±SD)**	111.64±36.89	0.040	0.574	0.098	0.174
**Triglycerides (mg/dL), Mean (±SD)**	142.18±72.41	0.022	0.763	0.065	0.369
**Metabolic syndrome**					
**Metabolic Syndrome**					
** No**	86 (43.9)	1.13±0.60	0.62	-0.39±1.04	0.35
** Yes**	110 (56.1)	1.20±1.23		-0.24±1.17	
**Atherosclerotic vascular disease (ASCVD) risk**					
**ASCVD 10 yrs risk (%), Mean (±SD)**	7.79±7.45	-0.133	0.154	-0.075	0.426
**Other**					
**CRP (mg/L), Mean (±SD)**	11.77±7.27	-0.006	0.934	-0.033	0.649
**Cortisol (μg/dL), Mean (±SD)**	18.47±12.27	-0.052	0.484	0.058	0.432

Bolded P-values are statistically significant as they are less or equal to 0.05.

Furthermore, when adjusting for age and gender, the association between C-peptide and ΔRTL remained significantly negative with a *P* value of 0.04 (**Table 3**). A similar trend was observed for both diabetes and C-peptide levels in association with RTL at follow-up, though not statistically significant.

**Table 3:** Multivariate linear regression showing association of diabetes related variables at baseline in 2014 with RTL at follow up in 2019 and change in RTL (ΔRTL) after adjustment for age and gender.

		95.0% Confidence Interval		
	β	Lower Bound	Upper Bound	P-value
	**RTL follow up**			
Definite Diabetes	-0.27	-0.651	0.108	0.16
C peptide (ng/dL)	-0.082	-0.187	0.024	0.12
	**ΔRTL**			
C peptide (ng/dL)	-0.11	-0.232	-0.002	0.04
Adjusted for age and gender				

Of note that we did not perform a sub-analysis on the group with diabetes because of small sample size (N=33) and because 17 of them (51.5%) were getting treatment with hypoglycemic medications. The cohort was hence relatively controlled with a mean ( ± SD) fasting serum glucose of 154 ± 47 (mg/dL), Insulin of 34.7 ± 13.0 (µIU/mL), HbA1c of 7.7 ± 1.8%, and C peptide of 3.8 ± 1.70 ng/dL.

**Figure 1:**
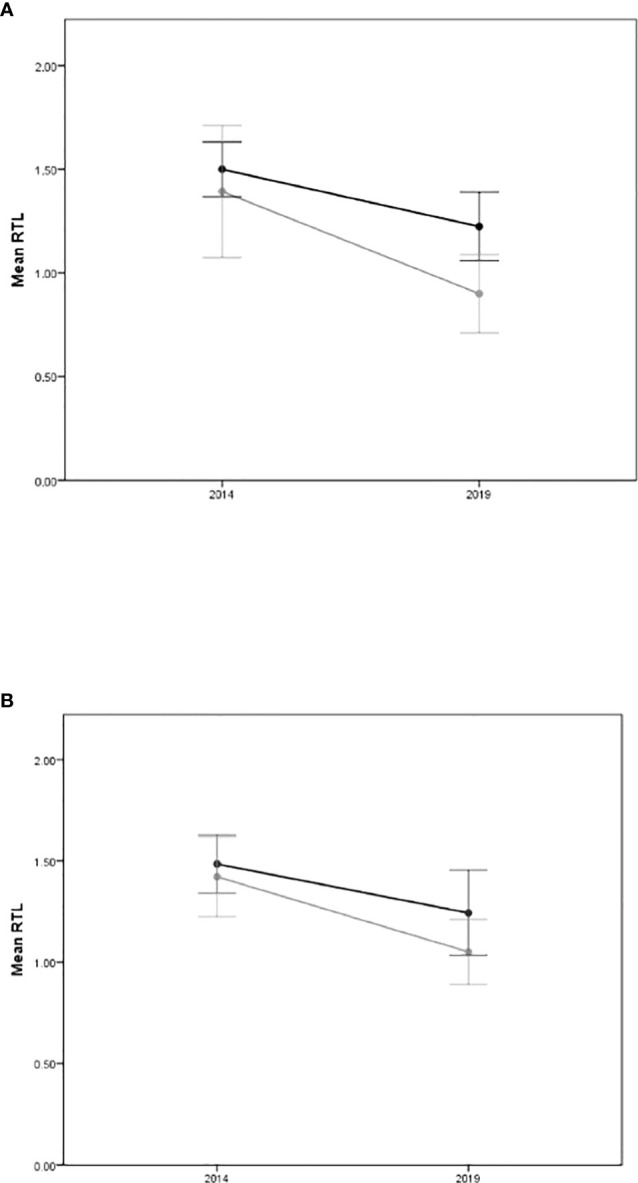
Mean RTL with 95% confidence intervals at baseline (2014) and at follow up (2019) for **(A)** definite diabetes status (Yes=grey line/No=dark line), and **(B)** C-Peptide levels (above the mean=grey line/below the mean=dark line).

**Figure 1** illustrates the change in RTL with diabetes (**A**) and C-peptide (**B**) for the whole cohort. It shows that participants with diabetes had a shorter RTL than participants without diabetes at baseline. RTL shortening occurred in both groups over the 5-year follow-up but was steeper in the group with diabetes (**Figure 1A**). Similarly, higher C-peptide levels (above the mean) were associated with shorter RTL at baseline. There was RTL shortening among all individuals but the group with higher C-peptide had a steeper drop over time (**Figure 1B**).

## Discussion

This study aimed to determine the relationship between baseline cardiometabolic risk factors, and telomere length over a 5-year period. Our results indicate that shorter RTL is significantly associated with higher C-peptide levels at baseline. A similar, though non-significant trend was observed for those who had diabetes at baseline. However, other risk factors such as hypertension and dyslipidemia did not show a statistically significant association with RTL at follow-up. To our knowledge, this is one of the very first studies to report on the long-term effect of metabolic derangement, and C-peptide in particular, on telomere length. This finding is of major clinical relevance as it may indicate how an individual’s health profile can influence RTL over the years, and therefore induce more rapid senescence. As a matter of fact, telomere loss is a widely described mechanism for cellular senescence, the irreversible loss of the cell’s replicative function, warranting its use as a marker for biological aging (22, 23).

Our results showed that older individuals had shorter telomere length at follow-up, a finding that is consistent with several studies (24–26). Furthermore, telomere length at follow-up was significantly associated with gender, with females having significantly longer RTL over the 5-year period. This is also consistent with the literature. A recent systematic review with meta-analysis concluded that females tend to have longer telomeres than males (27).

In the current study, our main finding is the longitudinal association whereby a higher C-peptide level predicted a shorter RTL and further shortening at 5-year follow-up. One other interesting, even though nonsignificant finding, is that the presence of diabetes at baseline was associated with a steeper drop in RTL over time. This sharper decrease in RTL (twice that of those who did not have diabetes at baseline) may imply accelerated aging, as seen in diabetes.

Our main finding on C-peptide is supported by a large cross-sectional survey conducted on 1382 adult men and women, where a longer telomere was associated with lower C-peptide level (28). Very few studies report on this association, and none on the longitudinal link. One possible mechanism by which C-peptide predicted RTL shortening may be through insulin resistance and hyperinsulinemia (29, 30). Telomere shortening has been reported to occur even before the onset of type 2 diabetes, at the prediabetes or even insulin resistance stage (31), consistent with our finding. Similarly, a community-based prospective study on 1382 participants from the NHANES population found that telomere length was inversely associated with C-peptide levels, independent of obesity and inflammation status (28). Moreover, in a subset of the Offspring cohort of the Framingham Heart Study, insulin resistance and oxidative stress were both found to be independently associated with shorter telomere length (32). Mechanisms linking insulin resistance with shorter telomere support oxidative stress (31), as the latter has been described to occur even before the development of hyperglycemia (33–35).

In this study, insulin levels did not associate with RTL, in parallel with C-peptide levels. This could be due to the inclusion of patients with diabetes, prediabetes and normoglycemia in our sample, a variability that may account for this lack of correlation. C-peptide is less dependent on glycemic variability (36), and studies have supported its use as a better marker for insulin resistance at the prediabetes and diabetes stage (37). In fact, C-peptide based indices have been shown to be better predictors of diabetes progression in the nondiabetic population (38). Moreover, in a study conducted in 2017, C-peptide levels were significantly higher in healthy individuals with insulin resistance than those without (39).This can be explained by the longer half-life of C-peptide compared to insulin (around five times higher) (40), and the fact that it is not metabolized by the liver (41), which provides more steady state levels for measurement (42), and more linear kinetics at the various glycemic stages. Alternatively, C-peptide may have effects independent of insulin (43), and is even hypothesized to have its own receptor (44).

Despite not reaching significance in our study, the link between diabetes and shorter RTL is better characterized in the literature than the one with C-peptide, and was shown across different populations, settings, and risk factors, in support of the trend found in our study (11, 45). *In vitro* studies show that hyperglycemia may have a direct effect on RTL shortening and subsequent cell senescence (46). This finding was demonstrated by an experiment where skin fibroblasts were cultured *in vitro* in a medium with high glucose concentration. As compared to a normoglycemic milieu, these fibroblasts reached the threshold for apoptosis within a shorter life cycle as measured by population doubling times. More importantly, the effect of hyperglycemia was circumvented by telomerase overexpression indicating telomere shortening can be reversed (46). One mechanism through which hyperglycemia may promote RTL shortening is through oxidative stress (47). The generated oxygen radicals affect telomere ends which are rich in guanine residues, the latter fact rendering them vulnerable to oxidative stress (11, 48, 49). The relation between diabetes, oxidative stress and telomere shortening has been described in various human cells such as fibroblasts (47), leukocytes (2), and more recently pancreatic beta cells (50). Oxidative damage in the latter could potentially lead to their senescence and further contribute to the decrease in insulin release and worsening of hyperglycemia (50), a relationship which may explain the bidirectional findings in studies on RTL and metabolic dysfunction (7).

In brief, a higher C-peptide and the presence of diabetes, are both associated with telomere shortening. Taken together, these risk factors are on a continuum of glucose metabolism, starting with insulin resistance, prediabetes, and diabetes. Our findings and those of the aforementioned studies indicate that biological aging could be influenced by these metabolic abnormalities. Given the prediabetes and diabetes epidemics, and the world increase in aging, and more specifically, premature aging, it becomes important and relevant to better characterize the link found in our study between elevated C-peptide and telomere shortening.

Our study suffers from some limitations. First, the small sample size for those who have definite diabetes (n=33) does not permit further exploration of diabetes control, duration of diabetes, and medication intake in relation to telomere shortening. In addition, the sample size may have been insufficient to show a statistically significant association between diabetes status and RTL at follow-up as well as between characteristic laboratory abnormalities in type 2 diabetes patients (HbA1c and fasting blood glucose) with change in RTL over time. In fact, even with regards to the significant association between C-peptide and RTL, the calculated power was only 55%. Furthermore, we looked at other cardiovascular risk factors such as obesity and smoking to evaluate whether there is a similar association as the one observed with diabetes, but the latter did not reach statistical significance, potentially due to the small sample size. Another limitation is that the participants were only recruited from the Greater Beirut area, which impedes its representativeness and warrants the need for further studies to explore the influence of diabetes on telomere length. Finally, telomere length was measured using quantitative PCR, a method described in 2009 by Cawthon (19). This method has some shortcomings including a low reliability for measurements at the bottom and top percentiles of RTL, as compared to the Southern blot technique, which is the gold standard method for estimating telomere length (51). However, the qPCR method is still widely used in most epidemiological studies (51).

Nonetheless, despite the limitations, the comprehensive evaluation of general risk factors is one of this study’s strengths. In fact, this reduces the risk of having any confounding bias that could affect the association between metabolic abnormalities and telomere length. Moreover, the follow-up period (5-year) was sufficient to observe the changes in telomere length over time. The consistency in measure and the fact that it is a prospective design are further unique strengths of this study.

## Data availability statement

The raw data supporting the conclusions of this article will be made available by the authors, without undue reservation.

## Ethics statement

The study was approved by the Institutional Review Board (IRB) at the American University of Beirut. The patients/participants provided their written informed consent to participate in this study.

## Author contributions

NZ conceived the idea. NZ, HT, RG designed the study. RG served as the primary author of the manuscript. RG, NZ and MN drafted the manuscript. HT and MM analyzed data. All authors contributed to the article and approved the submitted version.

## Funding

This work was supported by the AUB Medical Practice Plan (MPP).

## Conflict of interest

The authors declare that the research was conducted in the absence of any commercial or financial relationships that could be construed as a potential conflict of interest.

## Publisher’s note

All claims expressed in this article are solely those of the authors and do not necessarily represent those of their affiliated organizations, or those of the publisher, the editors and the reviewers. Any product that may be evaluated in this article, or claim that may be made by its manufacturer, is not guaranteed or endorsed by the publisher.

## References

[1] Shay JW. Telomeres and aging. Curr Opin Cell Biol (2018) 52:1–7. doi: 10.1016/j.ceb.2017.12.001 29253739

[2] Fasching CL. Telomere length measurement as a clinical biomarker of aging and disease. Crit Rev Clin Lab Sci (2018) 55(7):443–65. doi: 10.1080/10408363.2018.1504274 30265166

[3] Victorelli S Passos JF. Telomeres and cell senescence - size matters not. EBioMedicine (2017) 21:14–20. doi: 10.1016/j.ebiom.2017.03.027 28347656 PMC5514392

[4] Zhang J Rane G Dai X Shanmugam MK Arfuso F Samy RP. Ageing and the telomere connection: An intimate relationship with inflammation. Ageing Res Rev (2016) 25:55–69. doi: 10.1016/j.arr.2015.11.006 26616852

[5] Révész D Milaneschi Y Verhoeven JE Penninx BW. Telomere length as a marker of cellular aging is associated with prevalence and progression of metabolic syndrome. J Clin Endocrinol Metab (2014) 99(12):4607–15. doi: 10.1210/jc.2014-1851 25188715

[6] Zhao H Han L Chang D Ye Y Shen J Daniel CR. Social-demographics, health behaviors, and telomere length in the Mexican American mano a mano cohort. Oncotarget (2017) 8(57):96553–67. doi: 10.18632/oncotarget.19903 PMC5722504 29228552

[7] Elks CE Scott RA. The long and short of telomere length and diabetes. Diabetes (2014) 63(1):65–7. doi: 10.2337/db13-1469 24357701

[8] Révész D Milaneschi Y Verhoeven JE Lin J Penninx BW. Longitudinal associations between metabolic syndrome components and telomere shortening. J Clin Endocrinol Metab (2015) 100(8):3050–9. doi: 10.1210/jc.2015-1995 26133009

[9] Huzen J Wong LS van Veldhuisen DJ Samani NJ Zwinderman AH Codd V. Telomere length loss due to smoking and metabolic traits. J Intern Med (2014) 275(2):155–63. doi: 10.1111/joim.12149 24118582

[10] Verhulst S Dalgård C Labat C Kark JD Kimura M Christensen K. A short leucocyte telomere length is associated with development of insulin resistance. Diabetologia (2016) 59(6):1258–65. doi: 10.1007/s00125-016-3915-6 27020448

[11] Cheng F Carroll L Joglekar MV Januszewski AS Wong KK Hardikar AA. Diabetes, metabolic disease, and telomere length. Lancet Diabetes Endocrinol (2021) 9(2):117–26. doi: 10.1016/s2213-8587(20)30365-x 33248477

[12] Zgheib NK Sleiman F Nasreddine L Nasrallah M Nakhoul N Isma'eel H. Short telomere length is associated with aging, central obesity, poor sleep and hypertension in Lebanese individuals. Aging Dis (2018) 9(1):77–89. doi: 10.14336/ad.2017.0310 29392083 PMC5772860

[13] Mouneimne Y Nasrallah M Khoueiry-Zgheib N Nasreddine L Nakhoul N Ismail H. Bisphenol a urinary level, its correlates, and association with cardiometabolic risks in Lebanese urban adults. Environ Monit Assess (2017) 189(10):517. doi: 10.1007/s10661-017-6216-8 28942470

[14] Netzer NC Stoohs RA Netzer CM Clark K Strohl KP. Using the Berlin questionnaire to identify patients at risk for the sleep apnea syndrome. Ann Intern Med (1999) 131(7):485–91. doi: 10.7326/0003-4819-131-7-199910050-00002 10507956

[15] Nasrallah MP Nakhoul NF Nasreddine L Mouneimne Y Abiad MG Ismaeel H. PREVALENCE OF DIABETES IN GREATER BEIRUT AREA: WORSENING OVER TIME. Endocr Pract (2017) 23(9):1091–100. doi: 10.4158/ep171876.Or 28683240

[16] Armstrong C. JNC8 guidelines for the management of hypertension in adults. Am Fam Physician (2014) 90(7):503–4. 25369633

[17] Alberti KG Zimmet P Shaw J. The metabolic syndrome–a new worldwide definition. Lancet (2005) 366(9491):1059–62. doi: 10.1016/s0140-6736(05)67402-8 16182882

[18] Cawthon RM. Telomere measurement by quantitative PCR. Nucleic Acids Res (2002) 30(10):e47. doi: 10.1093/nar/30.10.e47 12000852 PMC115301

[19] Cawthon RM. Telomere length measurement by a novel monochrome multiplex quantitative PCR method. Nucleic Acids Res (2009) 37(3):e21. doi: 10.1093/nar/gkn1027 19129229 PMC2647324

[20] Pfaffl MW. A new mathematical model for relative quantification in real-time RT-PCR. Nucleic Acids Res (2001) 29(9):e45. doi: 10.1093/nar/29.9.e45 11328886 PMC55695

[21] Nasrallah MP Elbejjani M Nasreddine L Chami H Ismaeel H Fleifel M. Incidence of diabetes and its predictors in the greater Beirut area: a five-year longitudinal study. Diabetol Metab Syndr (2022) 14(1):67. doi: 10.1186/s13098-022-00833-w 35509100 PMC9066987

[22] Aubert G Lansdorp PM. Telomeres and aging. Physiol Rev (2008) 88(2):557–79. doi: 10.1152/physrev.00026.2007 18391173

[23] Bernadotte A Mikhelson VM Spivak IM. Markers of cellular senescence. telomere shortening as a marker of cellular senescence. Aging (Albany NY) (2016) 8(1):3–11. doi: 10.18632/aging.100871 26805432 PMC4761709

[24] Ehrlenbach S Willeit P Kiechl S Willeit J Reindl M Schanda K. Influences on the reduction of relative telomere length over 10 years in the population-based bruneck study: introduction of a well-controlled high-throughput assay. Int J Epidemiol (2009) 38(6):1725–34. doi: 10.1093/ije/dyp273 19666704

[25] Steenstrup T Hjelmborg JV Mortensen LH Kimura M Christensen K Aviv A. Leukocyte telomere dynamics in the elderly. Eur J Epidemiol (2013) 28(2):181–7. doi: 10.1007/s10654-013-9780-4 PMC3604590 23430034

[26] Berglund K Reynolds CA Ploner A Gerritsen L Hovatta I Pedersen NL. Longitudinal decline of leukocyte telomere length in old age and the association with sex and genetic risk. Aging (Albany NY) (2016) 8(7):1398–415. doi: 10.18632/aging.100995 PMC4993338 27391763

[27] Gardner M Bann D Wiley L Cooper R Hardy R Nitsch D. Gender and telomere length: systematic review and meta-analysis. Exp Gerontol (2014) 51:15–27. doi: 10.1016/j.exger.2013.12.004 24365661 PMC4523138

[28] Yang M Jiang P Jin C Wang J. Longer telomere length and its association with lower levels of c-peptide. Front Endocrinol (Lausanne) (2017) 8:244. doi: 10.3389/fendo.2017.00244 28959237 PMC5603756

[29] Mangge H Herrmann M Almer G Zelzer S Moeller R Horejsi R. Telomere shortening associates with elevated insulin and nuchal fat accumulation. Sci Rep (2020) 10(1):6863. doi: 10.1038/s41598-020-63916-6 32322021 PMC7176638

[30] Khan HA Sobki SH Ekhzaimy A Khan I Almusawi MA. Biomarker potential of c-peptide for screening of insulin resistance in diabetic and non-diabetic individuals. Saudi J Biol Sci (2018) 25(8):1729–32. doi: 10.1016/j.sjbs.2018.05.027 PMC6303159 30591792

[31] Gardner JP Li S Srinivasan SR Chen W Kimura M Lu X. Rise in insulin resistance is associated with escalated telomere attrition. Circulation (2005) 111(17):2171–7. doi: 10.1161/01.Cir.0000163550.70487.0b 15851602

[32] Demissie S Levy D Benjamin EJ Cupples LA Gardner JP Herbert A. Insulin resistance, oxidative stress, hypertension, and leukocyte telomere length in men from the framingham heart study. Aging Cell (2006) 5(4):325–30. doi: 10.1111/j.1474-9726.2006.00224.x 16913878

[33] Luc K Schramm-Luc A Guzik TJ Mikolajczyk TP. Oxidative stress and inflammatory markers in prediabetes and diabetes. J Physiol Pharmacol (2019) 70(6):809–24. doi: 10.26402/jpp.2019.6.01 32084643

[34] Evans JL Goldfine ID Maddux BA Grodsky GM. Oxidative stress and stress-activated signaling pathways: a unifying hypothesis of type 2 diabetes. Endocr Rev (2002) 23(5):599–622. doi: 10.1210/er.2001-0039 12372842

[35] Rani V Deep G Singh RK Palle K Yadav UC. Oxidative stress and metabolic disorders: Pathogenesis and therapeutic strategies. Life Sci (2016) 148:183–93. doi: 10.1016/j.lfs.2016.02.002 26851532

[36] Carr ALJ Oram RA Marren SM McDonald TJ Narendran P Andrews RC. Measurement of peak c-peptide at diagnosis informs glycemic control but not hypoglycemia in adults with type 1 diabetes. J Endocr Soc (2021) 5(10):bvab127. doi: 10.1210/jendso/bvab127 34377883 PMC8344843

[37] Chailurkit LO Jongjaroenprasert W Chanprasertyothin S Ongphiphadhanakul B. Insulin and c-peptide levels, pancreatic beta cell function, and insulin resistance across glucose tolerance status in thais. J Clin Lab Anal (2007) 21(2):85–90. doi: 10.1002/jcla.20138 17385686 PMC6649081

[38] Kim JD Kang SJ Lee MK Park SE Rhee EJ Park CY. C-Peptide-Based index is more related to incident type 2 diabetes in non-diabetic subjects than insulin-based index. Endocrinol Metab (Seoul) (2016) 31(2):320–7. doi: 10.3803/EnM.2016.31.2.320 PMC4923417 27349701

[39] Al Qarni AA Joatar FE Das N Awad M Eltayeb M Al-Zubair AG. Association of plasma ghrelin levels with insulin resistance in type 2 diabetes mellitus among Saudi subjects. Endocrinol Metab (Seoul) (2017) 32(2):230–40. doi: 10.3803/EnM.2017.32.2.230 PMC5503868 28555463

[40] Matthews DR Rudenski AS Burnett MA Darling P Turner RC. The half-life of endogenous insulin and c-peptide in man assessed by somatostatin suppression. Clin Endocrinol (Oxf) (1985) 23(1):71–9. doi: 10.1111/j.1365-2265.1985.tb00185.x 2863015

[41] Jones AG Hattersley AT. The clinical utility of c-peptide measurement in the care of patients with diabetes. Diabetes Med (2013) 30(7):803–17. doi: 10.1111/dme.12159 PMC3748788 23413806

[42] Sokooti S Kieneker LM Borst MH Muller Kobold A Kootstra-Ros JE Gloerich J. Plasma c-peptide and risk of developing type 2 diabetes in the general population. J Clin Med (2020) 9(9):3001. doi: 10.3390/jcm9093001 PMC7564789 32957570

[43] Ido Y. Diabetic complications within the context of aging: Nicotinamide adenine dinucleotide redox, insulin c-peptide, sirtuin 1-liver kinase B1-adenosine monophosphate-activated protein kinase positive feedback and forkhead box O3. J Diabetes Investig (2016) 7(4):448–58. doi: 10.1111/jdi.12485 PMC4931191 27181414

[44] Hills CE Brunskill NJ. Intracellular signalling by c-peptide. Exp Diabetes Res (2008) 2008:635158. doi: 10.1155/2008/635158 18382618 PMC2276616

[45] Wang J Dong X Cao L Sun Y Qiu Y Zhang Y. Association between telomere length and diabetes mellitus: A meta-analysis. J Int Med Res (2016) 44(6):1156–73. doi: 10.1177/0300060516667132 PMC5536737 28322101

[46] Blazer S Khankin E Segev Y Ofir R Yalon-Hacohen M Kra-Oz Z. High glucose-induced replicative senescence: point of no return and effect of telomerase. Biochem Biophys Res Commun (2002) 296(1):93–101. doi: 10.1016/s0006-291x(02)00818-5 12147232

[47] Araki E Nishikawa T. Oxidative stress: A cause and therapeutic target of diabetic complications. J Diabetes Investig (2010) 1(3):90–6. doi: 10.1111/j.2040-1124.2010.00013.x PMC4008021 24843413

[48] Ma D Zhu W Hu S Yu X Yang Y. Association between oxidative stress and telomere length in type 1 and type 2 diabetic patients. J Endocrinol Invest (2013) 36(11):1032–7. doi: 10.3275/9036 23873360

[49] Barnes RP Fouquerel E Opresko PL. The impact of oxidative DNA damage and stress on telomere homeostasis. Mech Ageing Dev (2019) 177:37–45. doi: 10.1016/j.mad.2018.03.013 29604323 PMC6162185

[50] Tamura Y Takubo K Aida J Araki A Ito H. Telomere attrition and diabetes mellitus. Geriatr Gerontol Int (2016) 16 Suppl 1:66–74. doi: 10.1111/ggi.12738 27018285

[51] Wang Y Savage SA Alsaggaf R Aubert G Dagnall CL Spellman SR. Telomere length calibration from qPCR measurement: Limitations of current method. Cells (2018) 7(11):183. doi: 10.3390/cells7110183 PMC6262465 30352968

